# The Peril of the University of Texas

**Published:** 1917-06

**Authors:** 


### EDITORIAL

### The Peril of the University of Texas.

If Governor Ferguson, and the men on the board who stand ready to do his bidding, can have their way, the University of Texas is doomed. While vitally interested in the entire University, the TEXAS MEDICAL JOURNAL is more directly concerned about the indirect attack being made on the Medical School at Galveston through the efforts to remove Dr. Thompson, and thus cripple that school. Not being able to find any ground for Dr. Thompson's removal, his ability being unquestioned, and taking advantage of the excitement over the war, a resolution was passed by the Board of Regents removing all aliens from the University as professors and instructors. Right then there was much discussion in the Legislature about "alien enemies" remaining in the employ of the State. To this there was no objection, but a joker was run into the resolution adopted by the Board of Regents of the University by leaving out the word "enemies." It was soon discovered that this resolution was a veiled effort to get Dr. Thompson out of the medical faculty, inspired by personal enmity, but it also was discovered that it was so broad in its scope that it would also remove a number of professors who have been with the main University for years and against whom there is no personal spite. To remove these professors, whose only sin is that they are natives of other countries that are now fighting with our country against Germany, would be such a manifest injustice that self-respecting Regents will not stand for it, so they have all been allowed to continue their work.

Dr. Thompson's ostensible offense is that he is an Englishman, who has never thought it important to complete his naturalization. He has been in this country many years, and has long ago made application for naturalization papers. He has been a voter for many years in Texas, and this JOURNAL is informed that he is subject to service for the country as a reserve naval medical officer. At any rate, his loyalty to the country and to the State is unquestioned, and no one can dispute his ability as a professor of surgery. If men are to be removed from public service because they are of English birth, why not carry it further and remove those of English descent? Were England at war with us the case would be different, but England is now at war with us and fighting for the same cause.

The action of Governor Ferguson and the members of the board under his domination in regard to Dr. Thompson and other faculty members shows that they are willing to destroy the University to build up their political fortunes or satisfy their personal spleen. No one doubts that if Dr. Thompson is finally removed, encouraged by their success, an effort will be made to remove Dr. Carter and perhaps other members of the medical faculty. The recent changes in the personnel of the board indicates clearly what may be expected in that respect, and it does not take a prophet to predict what will happen to the Texas Medical College, This college ranks as one of the best in the United States, and it is in the power of the physicians of the State to keep it so by showing the Board of Regents of the University in unmistakable terms that the medical profession of Texas is back of Drs. Thompson and Carter, and is unutterably opposed to political or personal prejudices dominating the school.

Physicians of the State, individually and collectively, should appeal to the Board of Regents to save the Medical School, for the removal of Drs. Thompson and Carter without cause will certainly prove its destruction. Men of lofty ideals will not subordinate their political views and personal freedom of thought to State employment, and a University professor who would do so is not worthy of a place in the institution.

